# Advances in Zosteric Acid: Antifouling Properties and Green Biosynthesis Toward Food Safety Applications

**DOI:** 10.3390/foods15050850

**Published:** 2026-03-03

**Authors:** Binghuang Zhu, Jie Zhou, Xiaomin Li

**Affiliations:** 1 State Key Laboratory of Food Science and Resources, Jiangnan University, Wuxi 214122, China; 1024230218@stu.jiangnan.edu.cn (B.Z.); 8202306008@jiangnan.edu.cn (J.Z.); 2 Key Laboratory of Industrial Biotechnology, Ministry of Education, School of Biotechnology, Jiangnan University, Wuxi 214122, China

**Keywords:** zosteric acid, antifouling, synthetic biology, microbial cell factory, 3′-phosphoadenosine-5′-phosphosulfate (PAPS)

## Abstract

Zosteric acid (ZA) is a marine-derived phenolic sulfate with great application potential as a natural antifouling agent in food safety. This review provides a comprehensive overview of ZA, focusing on its physio-chemical properties, structure–function relationships, and antifouling mechanisms, with particular emphasis on its relevance to controlling biofilms associated with food spoilage and foodborne contamination. It further discusses the major production strategies for ZA, including natural extraction, chemical synthesis, enzymatic synthesis, and microbial biosynthesis. Key challenges related to production efficiency, process scalability, and regulatory compliance are critically analyzed. Finally, future perspectives are proposed for the development and application of ZA in food processing, packaging, and hygiene control, with emphasis on integrating sustainable biosynthesis with practical implementation in food-related environments. This review aims to provide valuable insights that support the development of natural, safe, and sustainable antifouling strategies to enhance food safety and quality.

## 1. Introduction

Biofouling is an undesirable process resulting from the colonization and subsequent accumulation of biomass on submerged surfaces by unwanted living organisms, including animals, plants, and microorganisms [1]. In food-related environments, biofouling and biofilm formation on food-contact surfaces represent a critical challenge, as they directly compromise food hygiene, product quality, and consumer safety. In the food industry, microbial biofilms readily develop on processing equipment, pipelines, packaging materials, and storage surfaces, serving as persistent reservoirs for foodborne pathogens and spoilage microorganisms that are highly resistant to conventional cleaning and sanitization procedures. It also poses a major challenge for the biomedical and marine industries [2,3,4,5].

The presence of biofilms in food processing and storage systems not only increases the risk of cross-contamination and product recalls but also shortens shelf life and elevates economic losses across the food supply chain. To mitigate biofouling, a variety of physical and chemical strategies have been incorporated into antifouling (AF) coatings. Among them, metal ions and synthetic organic biocides, such as organo-mercury, dichlorobiphenyl-trichloroethane (DDT), and tributyltin (TBT), have been widely used due to their high efficacy [6,7,8]. However, the persistence and bioaccumulation of these compounds have been demonstrated to exert severe toxic effects on non-target organisms and ecosystems. From a food safety perspective, the potential migration of such toxic residues from treated surfaces into food products further restricts their practical application. Consequently, there is an urgent need for environmentally friendly antifouling alternatives that are effective yet nontoxic and biodegradable [9]. Natural antifouling agents derived from food-compatible or environmentally benign sources have therefore attracted increasing attention as promising candidates for next-generation food safety interventions. To this end, numerous sessile marine organisms, including algae, corals, starfish, and sponges, have been investigated as sources of natural secondary metabolites with antifouling activity [10]. Nevertheless, many marine-derived antifouling agents remain constrained by limited availability, difficulties in balancing activity and toxicity, insufficient stability and persistence under practical conditions, and an incomplete understanding of their mechanisms of action. These limitations have restricted their translation into scalable and safe solutions for food industry applications.

ZA, also known as *p*-(sulphonyl)cinnamic acid, is a naturally occurring phenolic sulfate isolated from seagrasses such as *Zostera marina* and *Zostera noltii*. ZA has emerged as one of the most extensively studied natural antifouling agents, exhibiting broad-spectrum antibiofilm activity against bacteria, fungi, algae, and other fouling organisms at sublethal concentrations [11]. Importantly, ZA has demonstrated strong antibiofilm efficacy against microorganisms that are highly relevant to food spoilage and foodborne contamination. Owning to its unique functional groups, ZA interferes with microbial adhesion and biofilm maturation, thereby reshaping biofilm architecture by reducing biomass, thickness, and structural integrity [12,13]. Beyond its antifouling performance, ZA possesses several characteristics that are particularly advantageous for food-related applications, including a simple molecular structure, high water solubility, low bioaccumulation potential, minimal ecotoxicity, strong biodegradability [14], and favorable cytocompatibility with both soft and hard tissues [15]. These properties render ZA a promising biocide-free antifouling agent for food-contact materials, food processing equipment, and active food packaging systems [16].

To date, ZA has been produced via four main routes: natural extraction from seagrasses, chemical synthesis, enzymatic synthesis, and microbial biosynthesis. However, natural extraction is impractical for industrial-scale production due to the low abundance of ZA in seagrass tissues and its dependence on seasonal and geographic factors [17]. Chemical synthesis can achieve high yields (up to ~95%) but relies on toxic sulfonating agents and costly precursors such as *p*-coumaric acid (*p*HCA), raising concerns regarding sustainability and food safety compliance. In contrast, enzymatic synthesis and microbial biosynthesis offer greener and potentially food-compatible alternatives, although challenges related to enzyme efficiency, metabolic burden, and process scalability remain.

Against this background, ZA represents a high-value bioactive compound with considerable potential in food safety, preservation, and hygiene control. This review systematically summarizes the physio-chemical properties and structure–activity relationships of ZA, with particular emphasis on its antifouling and antibiofilm functions. We further provide a comprehensive overview on ZA production strategies, highlighting recent advances in green biosynthesis enabled by metabolic engineering and synthetic biology, with the aim of supporting food safety, processing, and packaging applications. Finally, current challenges and future perspectives are discussed, focusing on the development of sustainable production platforms and the translation of ZA into practical food-related applications.

## 2. Structure–Function Relationships of ZA in Antifouling Activity

### 2.1. Physicochemical and Toxicological Properties

ZA is structurally derived from trans (E)-cinnamic acid, consisting of a phenyl ring connected via an ethenyl bridge to a carboxyl group (-COOH). A sulfate ester group (-OSO_3_H) is substituted at the para (4-) position of the aromatic ring. The conjugated π-electron system formed by the phenyl ring, the olefinic double bond, and the carbonyl group give rise to an extended planar structure [18], which has been associated with the antioxidant activity of ZA. The coexistence of the sulfate ester and carboxyl functionalities confers high water solubility and strong acidity, key physiochemical features underlying its biological performance in aqueous environments.

ZA is characterized by its ability to prevent microbial attachment and biofilm formation at non-toxic concentrations across a wide range of target organisms, including freshwater and marine bacteria, enterobacteria, yeasts, fungi, and complex environmental microbial communities, as comprehensively summarized by Kurth et al. [19]. Empirical studies have consistently demonstrated its potent antibiofilm activity under diverse conditions (Table 1). For example, Villa et al. reported that 500 mg/L ZA reduced surface coverage by bacterial biofilms (*Escherichia coli* and *Bacillus cereus*) by approximately 90% and fungal biofilms (*Aspergillus niger* and *Penicillium citrinum*) by 57% in vitro [12]. Similarly, Villa et al. showed that a much lower concentration (10 mg/L) reduced *Candida albicans* adhesion and subsequent biofilm formation by at least 70% [13]. The relatively low lethal concentration 50 (LC_50_) and effective concentration 50 (EC_50_) indicate that ZA achieves satisfactory antifouling performance against both gram-negative and -positive bacteria, as well as fungi, primarily through non-lethal mechanisms. Dose-dependent antifouling effects of ZA have also been observed in macrofouling organisms. In quagga mussels, concentrations below 250 mg/L were insufficient to prevent attachment, whereas concentrations up to 1000 mg/L effectively inhibited attachment during the first three days of exposure [20]. Importantly, ecotoxicological assays revealed no direct toxic effects of ZA toward *Daphnia magna* [15], underscoring its favorable environmental safety profile.

Beyond laboratory-scale studies, the antifouling efficacy of ZA has been validated in limited field trials. In one study, ceramic tiles coated with crude ZA and deployed in a marine environment for one week exhibited no barnacle attachment. In another field test, ZA was incorporated into a silicone-based foul-release coating and applied to immersion panels. After 60 days in seawater, these panels showed no hard fouling and significantly reduced slime fouling compared with control panels lacking ZA [21]. Together, these findings highlight the robustness of ZA’s antifouling performance under both controlled and environmentally relevant conditions.

**Table 1:** Antibiofouling performance towards target-organism.

Category	Organisms	Experimental Condition	Antibiofouling Performance	References
Gram-negative bacteria	*Pseudomonas putida*	[ZA]: 0~600 mg/L	200 mg/L ZA reduced bacterial coverage by 80%	[15]
		[ZA]: 5~500 mg/L	500 mg/L ZA reduced bacterial coverage by 98.2%	[22]
		[ZA]: 125, 250, 500 mg/L	EC_50_ = 167 ± 3.9 mg/L	[23]
	Lake Erie bacteria	[ZA]: 5~500 mg/L	50 mg/L ZA reduced bacterial coverage by 92.5%	[22]
		[ZA]: 5~500 mg/L	50 mg/L ZA reduced bacterial coverage by 90%	[24]
		[ZA]: 125, 250, 500 mg/L	EC_50_ = 375 ± 10 mg/L	[23]
	*Acinetobacter* sp.	[ZA]: 10^−1^~10^3^ μg/cm^2^	EC_50_ = 10 μg/cm^2^	[25]
	*Vibrio natriegens*	[ZA]: 5, 10 mg/L	EC_50_ = 7.4 mg/L	[23]
	*Vibrio parahaemolyticus*	[ZA]: 10, 20, 100 mg/L	EC_50_ = 18 mg/L	[23]
	*E*. *coli*	[ZA]: 0.183, 1.83, 18.3, 183, 1830 μmol/L	0.183 μmol/L is the minimum effective concentration	[26]
		[ZA]: 0, 10, 100, 200, 300, 400, and 500 mg/L	500 mg/L ZA reduced bacterial coverage by 90%	[12]
Gram-positive bacteria	*B*. *cereus*	[ZA]:0, 10, 100, 200, 300, 400, and 500 m/L	500 mg/L ZA reduced bacterial coverage by 90%	[12]
Fungi	*A*. *niger*	[ZA]: 0, 10, 100, 200, 300, 400, and 500 m/L	1000 mg/L ZA reduced fungi coverage by 57%	[12]
	*P*. *citrinum*	[ZA]: 0, 10, 100, 200, 300, 400, and 500 m/L	1000 mg/L ZA reduced fungi coverage by 57%	[12]
	*C*. *albicans*	[ZA]: 10 mg/L, added at different time point during biofilm formation	10 mg/L ZA reduced fungi coverage by 70%	[13]

### 2.2. Chemical Structure-Dependent Antifouling Efficacy

**Figure 1:**
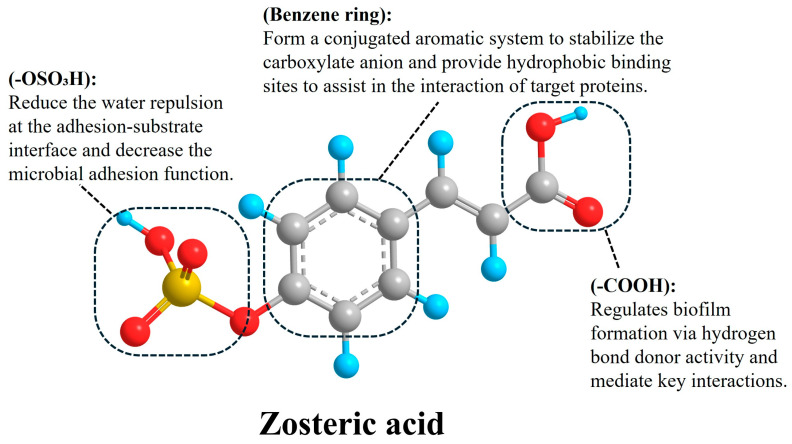
Structure–function–biosynthesis relationships of ZA. The gray balls represent carbon atoms; the blue balls represent hydrogen atoms; the red balls represent oxygen atoms; the yellow atom represents sulfur atom.

ZA contains three key structural elements: a sulfate ester group, a carboxyl group, and an aromatic π-conjugated system (Figure 1). Sulfated and sulfonated polymers have long been recognized for their ability to deter microbial adhesion and have been widely applied in antifouling membrane technologies [27]. Consistent with this concept, multiple studies have implicated the sulfate ester moiety of ZA in its antifouling performance, with EC_50_ values as low as 10 μg/cm^2^ against *Acinetobacter* sp., whereas their non-sulfated phenolic acid precursors were ineffective [25]. Similar conclusions were drawn by Stanley et al. in crop protection studies targeting fungal spore attachment [28] and by Callow et al. in investigations of *Enteromorpha* zoospores adhesion [29]. These studies collectively suggest that the sulfate group enhances surface hydration, thereby minimizing water exclusion at the adhesive–substratum interface and weakening adhesive interactions.

In contrast, Cattò et al. proposed that the aromatic system and carboxyl group play a dominant role in ZA’s antifouling activity [26]. Through the design and screening of a 43-member library of ZA scaffold derivatives against *E. coli*, they demonstrated that cinnamic acid (CA) itself reduced biofilm formation with a potency approximately 1000-fold greater than ZA. Conversely, derivatives in which the carboxyl group was replaced by an aldehyde or hydroxyl group showed negligible activity. These findings indicate that the carboxyl functionality—particularly its hydrogen-donating capability modulated by electronic effects from aromatic substituents—is critical for antifouling efficacy. Furthermore, the extended π-conjugated system associated with the trans-olefin configuration appears essential for stabilizing the carboxyl group and maintaining a bioactive molecular conformation. In this framework, the para-sulfate ester primarily enhances water solubility rather than serving as the principal antifouling determinant, a conclusion also supported by Kurth et al. [19].

Despite extensive investigation into the structure–function relationship of ZA, a critical reconciliation of the contradictory findings regarding the relative importance of the sulfate group and the carboxyl group remains lacking. This discrepancy primarily stems from the fragmentation of experimental systems and evaluation metrics. The “sulfate group dominance” perspective, derived mainly from early research on natural antifouling substances, correctly identifies the sulfate moiety as a key determinant of practical applicability [11,25]; it confers high water solubility, enhances surface hydration, and reduces hydrophobic interactions, which are essential for anti-adhesion in aqueous food processing environments. In contrast, the “carboxyl group dominance” viewpoint supported by pure culture and molecular docking studies highlights the carboxyl group as the biochemical pharmacophore [12,26,30], whose hydrogen-donating capability and interaction with microbial target proteins (e.g., WrbA) are responsible for the phenotypic modulation of biofilms. Holistic synthesis research reveals that these two groups act synergistically rather than competitively [11]. The carboxyl group defines the intrinsic potency of ZA against microbial biofilms, while the sulfate group dictates its bioavailability and environmental compatibility in real-world applications. The aromatic π-conjugated system serves as the structural scaffold that stabilizes the active conformation of the carboxyl group and modulates the electronic effect of the sulfate group. These conflicting reports reflect context-dependent functional priorities: the carboxyl group is indispensable for biological activity, whereas the sulfate group is irreplaceable for translating that activity into a food-safe, scalable solution. Future studies should move beyond single-group investigations and establish a four-dimensional structure–activity–application–safety model to guide the rational design of ZA-based antifouling materials.

From a synthetic biology standpoint, these structure–function insights have direct implications for pathway design and enzyme selection. In particular, the debated role of the sulfate ester group highlights the importance of sulfotransferase substrate specificity and catalytic efficiency in determining the final physicochemical properties of ZA. While the carbonylated aromatic scaffold appears to constitute the minimal antifouling pharmacophore, sulfation modulates solubility, diffusivity, and environmental compatibility—key parameters for real-world applications. Consequently, engineering biosynthetic routes that allow for precise control over sulfation patterns, either through enzyme engineering or pathway modularization, represents a central challenge and opportunity for the microbial production of ZA and its derivatives.

### 2.3. Antifouling Mechanisms of Action

Despite extensive investigation, the molecular mechanisms underlying ZA’s antifouling activity against bacterial and fungi biofilms have not been fully elucidated. Model-based analyses indicate that ZA does not function as a carbon or energy source, nor does it significantly affect microbial growth or surface wettability. Instead, ZA appears to modulate microbial behavior by increasing flagella abundance, motility, and chemotaxis, thereby reducing surface attachment and biofilm formation and enhancing chemotaxis [12]. Several non-exclusive mechanistic hypotheses have been proposed. These include (i) the alteration of interfacial water dynamics, minimizing water exclusion at the adhesive–substratum interface and reducing adhesive functionality [29]; (ii) the reduction in surface hydrophobicity on materials such as glass, polystyrene, and cellulose acetate, thereby weakening adhesive bonding [29]; (iii) in *C. albicans*, rapid reprograming of the yeast-to-hypha transcriptional network and disrupting transitions between planktonic (motile) and sessile (attached) states [16]; (iv) in *E. coli*, interaction with the quinone oxidoreductase WrbA, a putative biofilm modulator, triggering global stress response, enhanced flagella synthesis, and increased production of signaling molecules such as autoinducer-2 to facilitate escape from unfavorable environments.

ZA exhibits organism-specific modes of action. In bacteria, enhanced motility driven by increased flagellation reduces stable surface attachment. In fungi, antifouling effects may arise from interference with spore germination, adhesion, or growth, depending on the species. In algae, ZA has been reported to affect physiological processes such as algal photosynthesis and cell division. Collectively, these observations suggest that ZA functions primarily as a phenotypic modulator rather than a conventional biocide. Further mechanistic studies are required to bridge existing knowledge gaps and to fully exploit ZA’s potential as a versatile, non-toxic antibiofilm agent.

The antifouling efficacy of ZA is intrinsically linked to its distinct molecular structure, which governs its physicochemical properties and biological interactions at the biofilm–surface interface. These properties collectively enable ZA to modulate microbial adhesion and biofilm development without exerting overt cytotoxic effects. These structure–function insights have direct implications for pathway design and enzyme selection while also informing the rational development of ZA-based food-contact materials and antifouling surfaces.

## 3. Synthetic Biology-Driven Biosynthesis of ZA

**Figure 2:**
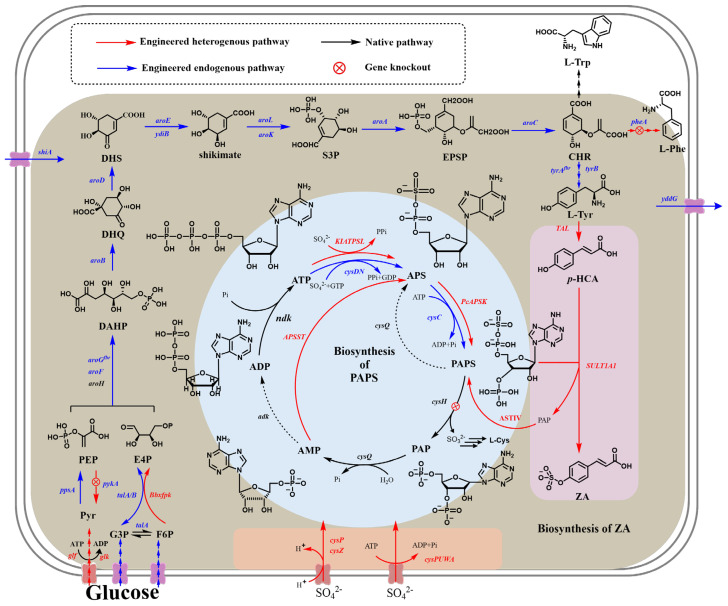
Modular synthetic biology design for *de novo* biosynthesis of ZA. Light yellow area represents the precursor supply module; light blue area represents the PAPS generation model; light orange area represents the sulfate transporting module; light purple area represents the sulfonation module. Arrows in black represent the native pathway in *E. coli*; and the dashed arrows represent the unproved step. Over-expression of endogenous proteins are indicated in blue, and heterologous proteins are indicated in red. The double gray boxes represent the cell membrane, and a cross in the red circle indicates gene knockout. Metabolite abbreviations: F6P, fructose 6-phosphate; G3P, glyceraldehyde-3-phosphate; PEP, phosphoenolpyruvate; Pyr, pyruvate; E4P, erythrose-4-phosphate; DAHP, 3-deoxy-D-arabino-heptulosonate-7-phosphate; DHQ, 3-dehydroquinate; DHS, 3-dehydroshikimic acid; S3P, shikimate 3-phosphate; EPSP, 5-enolpyruvylshikimate 3-phosphate; CHR, chorismic acid; L-Phe, L-phenylalanine; L-Trp, tryptophan; L-Tyr, L-tyrosine; PAP, 3′-phosphoadenosine 5’-phosphoadenosine; PAPS, 3′-phosphoadenosine 5’-phosphosulfate; APS, adenosine phosphosulfate; ATP, adenosine triphosphate; ADP, adenosine-5′-diphosphate; AMP, adenosine monophosphate; *p*HCA, *p*-hydroxycinnamic acid; ZA, zosteric acid. Protein abbreviations: Glf, *Z. mobilis*-derived UDP-galactopyranose mutase; Glk, glucokinase; Bbxfpk, *Bifidobacterium breve*-derived phosphoketolase; TAL, *Flavobacterium johnsoniae*-derived tyrosine ammonia-lyase; SULT1A1, *Rattus norvegicus*-derived sulfotransferase; KlATPSL, *Kluyveromyces lactis*-derived ATP sulfurylase; PcAPSK, *Penicillium chrysogenum*-derived adenosine 5′-phosphosulfate (APS) kinase; APSST, adenosine monophosphate sulfotransferase; ASTIV, aryl sulfotransferase; TalA, transaldolase; PpsA, phosphoenolpyruvate synthase; AroG/F/H, 3-deoxy-7-phosphoheptulonate synthase; AroB, 3-dehydroquinate synthase; AroD, 3-dehydroquinate dehydratase; YdiB, quinate/shikimate 5-dehydrogenase; AroE, shikimate dehydrogenase; AroL/K, shikimate kinase; AroA, 3-phosphoshikimate 1-carboxyvinyltransferase; AroC, chorismate synthase; TyrB, tyrosine aminotransferase; CysDN, ATP sulfurylase; CysC, APS kinase; CysQ, 3’(2’), 5′-bisphosphate nucleotidase; CysP, *Bacillus subtilis*-derived sulfate transporter; CysZ, CysZ sulfate transporter; CysPUWA, CysP-CysU-CysW-CysA sulfate ABC transporter complex; YddG, aromatic amino acid exporter; ShiA, shikimate and DHS importer; CysH, PAPS reductase; PykA, pyruvate kinase; PheA, chorismate mutase/pre phenate dehydratase; Adk, AMP kinase; Ndk, ADP kinase. Other abbreviations: Pi, phosphate.

With the development of synthetic biology, the most cost-competitive method for the complete synthesis of ZA from tyrosine or even glucose by microbial cell factories has been explored, which makes ZA an affordable AF agent. This approach follows a “design–build–optimize” paradigm, structured into four key modules: (i) sulfonation module; (ii) PAPS generation model; (iii) precursor supply module; and (iv) transporting module (Figure 2).

### 3.1. Sulfotransferase Discovery and Substrate Specificity

The feasibility of *de novo* ZA biosynthesis in microbial cell factories is fundamentally determined by the availability, regioselectivity, and catalytic performance of sulfotransferases (SULT) capable of mediating the site-specific sulfation of *p*HCA. To this end, SULTs originating from diverse biological sources—including the ZA-producing seagrass *Z. marina*, mammalian liver tissues, model organisms, and bacteria—have been systematically screened. Among the tested candidates, the cytosolic sulfotransferase SULT1A1 from *Rattus norvegicus* exhibited the highest catalytic efficiency toward *p*HCA and was therefore selected for heterologous expression in the *E. coli* and *Saccharomyces cerevisiae* chassis to construct ZA-producing strains [31]. SULTs represent a major design bottleneck due to their intrinsic biochemical properties. The sulfonation reaction is characterized by high substrate affinity but low catalytic capacity [32], and SULT activity is further constrained by competitive inhibition between the sulfate donor PAPS and the reaction by-product 3′-phosphoadenosine-5′-phosphate (PAP) [33]. Structural studies have revealed the formation of dead-end complexes, including PAPS-SULT-receptor and PAP-SULT-product assemblies, which severely limit turnover [34]. To address these limitations, directed evolution strategies have been employed to enhance both the activity and thermostability of SULT [35]. In addition, a site-mutated rat sulfotransferase IV (ASTIV) was introduced to regenerate PAPS by transferring a sulfuryl group from *p*-nitrophenyl sulfate (*p*NPS) to PAP, thereby alleviating the PAP-mediated inhibition of SULT [36,37].

Collectively, these findings highlight sulfotransferase specificity and robustness as the primary gatekeepers of ZA biosynthesis, making enzyme discovery and engineering indispensable starting points for synthetic biology-driven sulfate ester production.

### 3.2. PAPS Metabolism and Sulfur Donor Economy

Beyond enzyme specificity, the intracellular economy of the sulfur donor (PAPS) emerges as the central bottleneck governing flux through the sulfonation module of the ZA biosynthetic pathway. As the universal sulfuryl donor in biological sulfation reactions, PAPS availability has been identified as the primary bottleneck in ZA biosynthesis. In native host metabolism, PAPS is synthesized from sulfate and ATP via the sequential actions of ATP sulfurylase (CysD/CysN) and APS kinase (CysC), but it is also rapidly consumed by the competing cysteine synthesis pathway through PAPS reductase (CysH).

To enhance intracellular PAPS levels, several engineering strategies have been implemented. Overexpression of the endogenous *cysDNC* operon or heterologous combinations of ATP sulfurylase and APS kinase enzymes (e.g., *Kl*ATPSL, *Pc*APSK, and *Bs*CYSP), coupled with the deletion of *cysH*, effectively blocked PAPS reduction and resulted in more than a 1000-fold increase in PAPS accumulation [38]. However, excessive manipulation of sulfur metabolism introduces a significant energetic burden, as PAPS synthesis consumes two equivalents of ATP per molecule. An additional challenge arises from PAP accumulation. While the endogenous phosphatase CysQ converts PAP to AMP and promotes PAP clearance, it has also been shown to hydrolyze PAPS to APS, leading to futile cycling and energy dissipation. Consequently, the overexpression of CysQ paradoxically reduces overall *p*HCA and ZA production [38], underscoring the delicate balance required in the sulfur donor economy.

More recently, Gu et al. proposed an artificial PAPS biosynthesis pathway that utilizes AMP instead of ATP as the immediate precursor, combined with an AMP supplementation module based on ribose and adenosine [39]. This design decouples PAPS from ATP-intensive central metabolism, illustrating how synthetic biology can rewire cofactor usage to resolve resource conflicts in complex biosynthetic networks. Optimizing sulfur donor metabolism is critical for improving ZA productivity and ensuring process feasibility for food-related applications.

### 3.3. Precursor Supply and Central Carbon Metabolism Reprogramming

An efficient precursor supply for ZA biosynthesis relies on the robust supply of the aromatic precursor *p*HCA and careful balancing of *p*HCA production with central carbon metabolism, as the excessive accumulation of *p*HCA imposes energetic stress and compromises host growth.

In engineered *E. coli*, a heterologous tyrosine ammonia lyase (TAL) from *Flavobacterium johnsoniae* has been introduced to catalyze the non-oxidative deamination of L-tyrosine to *p*HCA [37]. Alternatively, *p*HCA can also be synthesized via the phenylalanine ammonia-lyase (PAL)-mediated conversion of L-phenylalanine to trans-cinnamic acid, followed by hydroxylation by trans-cinnamate-4-monooxygenase [40]. To maximize precursor availability, extensive rewiring of the shikimate pathway has been performed. Feedback-resistant variants of DAHP synthases AroF^fbr^ and AroG^fbr^ were overexpressed to enhance flux into aromatic amino acid biosynthesis, while the deletion of the transcriptional regulator *tyrR* relieved the repression of genes involved in phenylalanine and tyrosine formation [41,42,43]. Precursor pools of phosphoenolpyruvate (PEP) and erythrose-4-phosphate (E4P) were further increased through the deletion of *pykA* and the *ptsHIcrr* operon to conserve PEP, along with the overexpression of heterologous *fbk* to boost the E4P supply from the pentose phosphate pathway. The downstream enzymes TyrA and TyrC were also engineered for feedback resistance and overexpression to reinforce L-tyrosine biosynthesis [31,37].

Despite these optimizations, the excessive accumulation of *p*HCA (>0.4 mg/mL) imposes significant metabolic stress, reducing the intracellular ATP pool and thus hindering cell growth [44]. This imbalance often leads to acetate overflow during early fermentation stages, indicative of disrupted central metabolism. Moreover, *p*HCA accumulation exacerbates PAPS deficiency, potentially due to ATP depletion or its structure’s similarity to phenolic uncouplers like 2,4-dinitrophenol [45]. These observations highlight the necessity of balancing precursor production with sulfonation capacity and cellular energy metabolism. To maintain ATP generation, the selective deletion of *pykA* while retaining *pykF* was shown to preserve flux into the TCA cycle and conserve PEP for aromatic acid biosynthesis, thereby supporting cell growth and ZA production, respectively [38]. Alternatively, strain robustness has been enhanced through stress-induced mutagenesis-based adaptive laboratory evolution (ALE) [46], combined with biosensor-assisted titratable CRISPRi high-throughput (BATCH) screening [47], yielding strains with improved tolerance to ZA and its intermediates. Therefore, precursor engineering for ZA production must move beyond maximizing aromatic flux and instead adopt a balanced strategy that preserves energy homeostasis and growth-coupled productivity.

During fermentation with the ZA-producing strain, the precursor *p*HCA is found to be degraded into a by-product 4-hydroxystyrene, probably under the catalysis of phenolic acid decarboxylase (PAD). However, there is no gene annotated to encoding PAD in the genome of the *E. coli* host, nor any protein sequence aligning with the homologous PAD in the proteome. As the enzyme that performs PAD activity in *E. coli* is not yet known, there is not any metabolic strategy to inhibit *p*HCA degradation but to add the *p*HCA inhibitor benderizine hydrochloride (BH). These strategies demonstrate the potential of biosynthetic approaches to supporting the sustainable production of sulfate esters while meeting food safety and environmental requirements.

### 3.4. Transport Systems and Cellular Homeostasis

Transport processes and cellular homeostasis represent an additional, often underappreciated, constraint in ZA production, influencing substrate uptake efficacy, intermediate conservation, and product export. As a result, transport engineering constitutes the final design layer in ZA-producing cell factories.

To reduce PEP consumption associated with glucose uptake, the native phosphotransferase system (PTS) has been replaced with a facilitated diffusion system comprising glucose facilitator Glf and glucokinase Glk. Transcriptomic analyses revealed that the co-expression of SULT1A1 and *cysDNC* induces sulfate starvation, suggesting that enhanced sulfate uptake may further improve ZA biosynthesis. Accordingly, the overexpression of sulfate transporters such as the *cysPUWA* ABC transporter or *Bacillus subtilis* CysP has been explored under conditions of high sulfate demand. However, the excessive expression of membrane transporters often compromises membrane integrity and cellular fitness, necessitating careful tuning via low-copy plasmids. To minimize intermediates loss, the shikimate importer ShiA was overexpressed to recapture leaked shikimate, resulting in moderate improvements in ZA production. Given that L-tyrosine feeding can enhance ZA titers [38], the aromatic amino acid transporters (*tyrP*, *aroP*) and the exporter *yddG* were individually or combinatorially expressed to modulate import and export fluxes [37]. Unexpectedly, these interventions yielded limited benefits, potentially due to regulatory interference with tyrosine-sensitive genes. Although ZA is ultimately secreted into the culture supernatant, the molecular governing of the transport of ZA and other sulfated aromatic compounds remains largely unexplored. Elucidating these transport processes represents an important frontier for achieving predictable secretion, reducing intracellular stress, and enabling the scalable microbial production of sulfated metabolites.

Together, these observations underscore transport and cellular tolerance as critical yet insufficiently understood layers of ZA biosynthesis, warranting further investigation to achieve robust and scalable production.

### 3.5. Coupling of Biosynthetic Modules at the Systems Level

While precursor supply, sulfonation, cofactor metabolism, and transport are often engineered as discrete modules, ZA biosynthesis ultimately emerges from their tight functional coupling within the cellular system. Perturbation in one module may rapidly propagate to others, manifesting as energy stress, growth inhibition, or flux collapse. First, the accumulation of the sulfur donor PAPS is tightly coupled with precursor metabolism. In the engineered *E. coli*, the sulfonation rate of ZA decreased progressively with increasing *p*HCA levels as it exceeded 0.5 mM; with 3.0 mM supplemented *p*HCA, the sulfonation rate of ZA dropped sharply to as low as 4.4%, whereas 60% of pHCA remained unsulfurated. These results indicate that excessive precursor accumulation indirectly hinders PAPS synthesis by inhibiting ATP generation, leading to cross-module flux suppression [37]. Second, PAPS deficiency directly inactivates the sulfonation module. It has been proven that strains with the deleted PAPS-degrading gene *cysH* and overexpressed PAPS-synthesizing genes *KlATPSL* and *PcAPSK* achieved a ZA titer of 11.40 mM in fed-batch fermentation supplemented with 15 mM L-tyrosine, with no unsulfurated *p*HCA detected. In contrast, strains without *cysH* deletion produced only 0.73 mM ZA under the same conditions, with a concomitant accumulation of 9.0 mM *p*HCA, corresponding to a sulfonation efficiency of less than 8%; and strains with only PAPS-synthesis-related genes *cysDN* and *cysC* overexpression yielded less than half the ZA produced by strains with both engineering manipulations [37]. These findings confirm that the optimization of the sulfur donor synthesis module must be coordinated with precursor metabolism; otherwise, even with sufficient precursors, sulfonation flux collapses due to PAPS limitation. Consequently, successful ZA production requires coordinated optimization across enzyme specificity, sulfur donor economy, central carbon metabolism, and cellular homeostasis rather than isolated pathway amplification.

ZA production in microbial cell factories is constrained by four interconnected modules: (A) sulfotransferase specificity governing the regioselective sulfonation of *p*HCA; (B) PAPS cofactor economy and energy-intensive sulfur activation; (C) precursor supply and metabolic flux balance between growth and production; and (D) transport and cellular homeostasis, including substrate uptake, intermediate leakage, and product export. From a food production perspective, the development of microbial cell factories for ZA biosynthesis requires the coordinated control of precursor supply and cofactor availability to enable the sustainable and scalable production of food-grade ZA (Table 2).

Overall, ZA serves as a representative example of how green biosynthesis can enable the sustainable and food-compatible production of natural antifouling agents with relevance to food safety, processing, and packaging.

## 4. Exploration of Main Application of ZA

As a naturally synthesized sulfated phenolic acid, the key advantage of ZA lies in its non-biocidal anti-adhesion/anti-biofilm activity: it inhibits the initial adhesion and biofilm formation of bacteria, fungi, algae, and marine fouling organisms without directly killing microorganisms. It also features low toxicity, high water solubility, ease of chemical synthesis and microbial fermentation production, and environmental friendliness, providing a new route to replace traditional, highly toxic functional chemicals. Although ZA has not yet been commercialized on a large industrial scale, pilot-scale verification and small-scale tests have been completed in several fields, establishing clear application directions and technical pathways.

### 4.1. Application in Marine Antifouling and Anti-Biofouling

Marine biofouling (e.g., attachment of barnacles, algae, and microorganisms on ship hulls, marine pipelines, offshore platforms, etc.) significantly increases navigation energy consumption, accelerates equipment corrosion, and shortens service life. Traditional antifouling technologies mostly rely on antifoulants containing toxic components such as copper and organotin, causing severe damage to the marine ecological environment. The non-biocidal anti-adhesion properties of ZA make it a key candidate in green marine antifouling research. Pilot-scale production verification and small-scale application tests have been completed.

The EU Horizon 2020 FTI project ZABIO is the core platform for the industrialization of ZA in this field, jointly implemented by Cysbio, Bio Base Europe Pilot Plant (BBEPP), and Henkel. It focuses on the large-scale microbial fermentation production of ZA and its application in marine antifouling. BBEPP participated in the pilot-scale microbial fermentation and purification process development of ZA. After completing pilot-scale fermentation and purification, BBEPP delivered bulk high-purity ZA samples to Henkel for antifouling product testing. All pilot tasks were completed from 2020 to 2023.

Related tests mainly focused on the development of antifoul coatings and sealants, aiming to produce long-lasting antifouling coatings for ship materials and marine pipeline surfaces to inhibit microbial adhesion and biofilm formation with no obvious cytotoxicity, meeting non-biocidal antifouling requirements.

### 4.2. Application Potential in Medical Healthcare

Biofilm formation on the surfaces of medical implants (e.g., artificial joints, cardiac stents), catheters, and wound dressings is a major cause of clinical infections. Traditional antibacterial materials mostly use biocidal agents, which may induce bacterial resistance and adverse biological reactions. The anti-biofilm activity and low toxicity of ZA have attracted extensive attention in medical material modification.

Laboratory studies have confirmed the broad-spectrum anti-biofilm potential of ZA at sublethal concentrations by interfering with the quorum-sensing system of pathogenic bacteria, inhibiting the initial adhesion and maturation of biofilms rather than directly killing bacteria, and, finally, effectively reducing the generation of drug-resistant strains [26]. Therefore, immobilizing ZA on medical material surfaces via embedding, covalent grafting, or other strategies is expected to inhibit biofilm formation by common pathogens, including *Staphylococcus aureus*, *E*. *coli*, and *C. albicans*, etc.

### 4.3. Application Potential in Food Safety

#### 4.3.1. Crop Protection

Traditional agricultural crop protection relies on biocidal fungicides. Long-term use often leads to pathogen resistance, soil and water pollution, and pesticide residues in agricultural products. Early research in phytopathology confirmed that ZA effectively inhibits the spore adhesion of *Magnaporthe grisea* and *Colletotrichum lindemuthianum*, and low-concentration treatment significantly reduces crop lesions, laying the foundation for its application in non-biocidal crop protection [28].

Its application potential in crop protection has been preliminarily verified on a laboratory scale, which showed that ZA controls plant diseases by inhibiting spore adhesion and infection of phytopathogens, with no toxicity to crops or beneficial microorganisms, matching the requirements of green agriculture. However, no mature technical system supporting patent layout has been formed; therefore, technical reserves such as formulation and application methods still require improvement. With further laboratory research, pilot trials, and data accumulation, related technologies are expected to complete patent filing and layout, providing technical support for future industrial manufacture and pesticide registration.

#### 4.3.2. Food Processing

Microbial adhesion on food processing equipment and food packaging materials would raise the risks of food contamination. Based on its anti-biofilm anti-adhesion properties, ZA can be applied in the modification of food processing pipelines and packaging materials to inhibit microbial adhesion, with low toxicity, thus meeting safety standards for food-contact materials. Although current research remains in the laboratory stage, focusing on optimizing immobilization methods and long-term effectiveness is expected to lay a theoretical foundation for upcoming applications.

#### 4.3.3. Industrial Water Treatment

In industrial water treatment systems, biofilm formation on membranes (e.g., MBR membranes) and pipeline inner walls causes membrane fouling, flux decline, and pipe blockage, increasing cleaning frequency, chemical disinfectant usage, and treatment costs. The application of ZA in water treatment is also expected to be realized, relying on its non-biocidal anti-biofilm and anti-adhesion capabilities.

Among these above fields, only marine antifouling has established traceable pilot production and small-scale testing technical pathways. Specifically, microbial fermentation pilot tests (with BBEPP) and joint tests with Henkel have been completed, making it the most commercially mature direction. Applications of ZA in medical healthcare and food safety, such as agricultural crop protection, industrial water treatment, and food processing are still at the preliminary laboratory exploration stage, lacking publicly traceable pilot applications and requiring further research and data accumulation.

## 5. Challenges, Limitations, and Future Perspectives

Despite the considerable antifouling and antibiofilm potential of ZA, several challenges and limitations must be addressed before its broader practical application can be realized. From a food industry perspective, these challenges extend beyond bioactivity and production efficiency, encompassing food safety compliance, material compatibility, and regulatory acceptance.

One of the primarily limitations associated with ZA is its current production scalability (Table 2). Natural extraction from seagrasses is inherently unsustainable due to low yields, seasonal variability, and ecological concerns. Chemical synthesis, although capable of achieving high yields, relies on toxic sulfonating reagents and non-renewable precursors, raising concerns regarding environmental impact and downstream purification. For food-related applications, the use of hazardous chemicals further limits the acceptability of chemically synthesized ZA due to the potential risk of residual contaminants and regulatory restrictions. In contrast, enzymatic synthesis and microbial biosynthesis represent greener alternatives; however, challenges such as limited enzyme stability, insufficient cofactor availability, metabolic burden, and suboptimal titers remain to be overcome.

Another critical challenge lies in the translation of ZA from laboratory-scale studies to real-world applications. Although ZA has demonstrated strong antibiofilm activity at sublethal concentrations, its long-term efficacy and stability under complex operational conditions require further investigation. In food processing environments, antifouling agents must withstand repeated cleaning-in-place (CIP) procedures, temperature fluctuations, mechanical stress, and prolonged exposure to moisture and organic matter. The performance of ZA under such conditions, particularly when immobilized on food-contact materials, remains largely unexplored.

In addition, while ZA is generally regarded as environmentally benign, its safety profile in food-related contexts warrants more systematic evaluation. Comprehensive toxicological assessments, including chronic exposure studies, migration behavior from coated surfaces into food simulants, and potential impacts on sensory attributes of food products, are essential to supporting regulatory approval. Establishing clear structure–function–safety relationships will be critical for positioning ZA as a viable antifouling agent in food packaging and processing systems.

Looking forward, several research directions offer promising opportunities to advance ZA toward food-related applications. The integration of metabolic engineering and synthetic biology provides a powerful platform for meeting food safety, regulatory, and sustainability requirements. The further optimization of microbial cell factories, including precursor supply, cofactor regeneration, and transporter engineering, is expected to improve productivity and cost-effectiveness.

From an application standpoint, future efforts should focus on incorporating ZA into food-contact materials, active food packaging, and surface coatings designed for food processing equipment. Strategies such as controlled release systems, covalent immobilization, and hybrid material design may enhance the durability and functionality of ZA-based antifouling surfaces. Importantly, combining antifouling performance with food preservation and hygiene control could enable multifunctional material systems that simultaneously inhibit biofilm formation and extend food shelf life.

In conclusion, ZA represents a promising natural antifouling agent with emerging relevance to food safety, processing, and packaging applications. Addressing current challenges related to sustainable production, material integration, and regulatory compliance will be essential for its successful translation from laboratory research to industrial practice. Continued interdisciplinary collaboration among food scientists, microbiologists, material scientists, and synthetic biologists will play a key role in unlocking the full potential of ZA within the food sector.

## 6. Conclusions

ZA has emerged as a promising natural antifouling and antibiofilm agent with broad-spectrum activity against diverse fouling microorganisms. This review summarized the physio-chemical properties, structure–activity relationships, antifouling mechanisms, and production strategies of ZA, with a particular focus on recent advances in green biosynthesis enabled by metabolic engineering and synthetic biology. By integrating fundamental insights with application-oriented perspectives, this review highlights the growing relevance of ZA beyond marine and biomedical contexts, extending into food-related environments. Continued advances in enzyme engineering, cofactor management, modular pathway integration, and host strain robustness are expected to enable cost-effective and industrially viable ZA production. Combining antifouling functionality with food preservation and shelf-life extension represents a particularly promising direction for the development of next-generation multifunctional food materials, thus successfully translating ZA into real food-related applications. The insights provided in this review are expected to support the development of safe, sustainable, and effective antifouling solutions that contribute to a more resilient and hygienic food system.

## Figures and Tables

**Table 2:** Comprehensive comparison of ZA production strategies.

Evaluation Dimension	Natural Extraction	Chemical Synthesis	Enzymatic Synthesis	Microbial Synthesis
Core principle	Isolation and purification are conducted for marine plants, including *Z*. *marina* and *Z*. *noltii* [17].	O-sulfonation reaction is performed using *p*HCA as the substrate and reagents such as chlorosulfonic acid [11].	Sulfotransferaseor arylsulfotransferase is used as the biocatalyst to catalyze the O-sulfonation of p-coumaric acid with PAPS as the sulfate donor.	*E*. *coli* or *S*. *cerevisiae* is genetically modified by introducing TAL and SULT, followed by the *de novo* synthesis of ZA using glucose, glycerol, or tyrosine as raw materials [31,37].
Yield	Extremely low: 1. Content in plant dry weight: 51–692 μg/g. 2. Titer of extraction broth: <0.1 g/L. 3. Batch scale: Milligram level.	Medium: 1. Laboratory batch scale: Gram level. 2. Purification yield: 30–50%. 3. Presence of multiple side reactions and poor selectivity.	Medium to high: 1. Laboratory batch scale: Gram to decagram level. 2. Catalytic yield: 60–85%. 3. High substrate selectivity, minimal side reactions.	High: 1. Fed-batch fermentation of *E*. *coli*: 1.52 g/L. 2. Fed-batch fermentation of *S*. *cerevisiae*: 5 g/L. 3. Scalable to a 100-ton-level fermentation scale.
Scalability	Extremely poor: 1. Dependent on wild or cultivated *Zostera*, which is limited by sea area, season, and climate. 2. Complex extraction and purification processes, making continuous scale-up difficult.	Medium: 1. Harsh reaction conditions (low temperature, anhydrous environment, strong corrosiveness). 2. High safety and environmental pressure after scale-up. 3. Significant increase in purification cost with the expansion of scale	Medium: 1. Mild reaction conditions, low safety and environmental pressure. 2. Limited by the high cost and low availability of PAPS and purified enzymes. 3. Difficult to maintain enzyme activity during large-scale continuous catalysis.	Excellent: 1. Based on standard fermentation processes, it can be directly scaled up from shake flasks to a 100-ton level. 2. Raw materials are bulk carbon sources with a stable supply chain. 3. Single product and mature, scalable purification processes
Cost	Extremely high: 1. High cost of raw material collection and transportation. 2. Low extraction efficiency and high solvent consumption. 3. Multiple purification steps. 4. Estimated cost: >USD 10,000/kg.	High: 1. Expensive and hazardous reagents (e.g., chlorosulfonic acid, pyridine). 2. High reaction energy consumption and high cost of three-waste (waste gas, waste water, solid waste) treatment. 3. Low purification yield leading to increased unit cost.	High: 1. High cost of purified enzymes and PAPS. 2. Additional cost for enzyme immobilization (to improve reusability). 3. Estimated cost: USD 5000–8000/kg.	Low: 1. Raw materials are glucose or glycerol with a cost of <USD 1/kg. 2. Low fermentation energy consumption and few by-products. 3. Estimated cost after scale-up: <USD 100/kg.
Food-grade feasibility	Feasible but with limitations: 1. Natural source without residual chemical reagents. 2. Raw materials are susceptible to contamination by marine heavy metals and microplastics. 3. Poor batch stability, which is difficult for meeting food-grade purity requirements.	Basically unfeasible: 1. Utilization of strongly corrosive and toxic reagents, which are prone to residue. 2. Complex structure of by-products, making complete removal difficult. 3. Failure to meet food contact or edible safety standards.	Feasible but limited: 1. Biocatalysts are biodegradable, with no toxic reagent residues. 2. High-cost limits large-scale food-grade application. 3. PAPS residue may affect food safety compliance.	Optimal: 1. Chassis can be selected from GRAS strains (e.g., *S*. *cerevisiae*). 2. Mild reaction conditions without toxic reagents. 3. Purity can easily reach 99% or higher, achievable through food-grade purification processes. 4. Risks such as endotoxins and heavy metals can be eliminated through metabolic engineering, meeting the food additive standards of FDA and EFSA.
Applicable scenarios	Small-batch, high-value-added, non-food scenarios.	Non-food, cost-insensitive industrial or pharmaceutical intermediates.	Non-food, high-purity-required industrial or pharmaceutical intermediates.	Industrial production and food-grade applications.

## Data Availability

No new data were created or analyzed in this study. Data sharing is applicable to this article.

## Abbreviations

The following abbreviations are used in this manuscript:

ZA	Zosteric acid
AF	Antifouling
TBT	Tributyltin
*p*HCA	*p*-Coumaric acid
CA	Cinnamic acid
SULT	Sulfotransferases
PAPS	3′-phosphoadenosine-5′-phosphosulfate
PAP	3′-phosphoadenosine-5′-phosphate
ASTIV	A site-mutated rat sulfotransferase IV
*p*NPS	*p*-Nitrophenyl sulfate
TAL	Tyrosine ammonia lyase
PEP	Phosphoenolpyruvate
E4P	Erythrose-4-phosphate
ALE	Adaptive laboratory evolution
BATCH	Biosensor-assisted titratable CRISPRi high-throughput
PAD	Phenolic acid decarboxylase
BH	Benderizine hydrochloride
PTS	Phosphotransferase system
BBEPP	Bio Base Europe Pilot Plant
